# Supplementary material for: MetaRibo-Seq measures translation in microbiomes
Source: Nat Commun. 2020 Jun 29;11:3268. doi: 10.1038/s41467-020-17081-z (PMC7324362; doi:10.1038/s41467-020-17081-z)
Supplement: Supplementary file 10 — Supplementary Data 7 [file 41467_2020_17081_MOESM10_ESM.zip › File2/Confidence_High_Taxonomy/290639_out.krona.html]

Javascript must be enabled to view this page.

members
magnitude
magnitudeUnassigned
count
unassigned
taxon
rank

290639\_out

81

2
superkingdom
81

1239
phylum
81

186801
81
class

order
81
186802

81
family
186806

81
genus
1730


SRS011084\_contig\_number\_34136SRS012273\_contig\_number\_13877SRS013940\_contig\_number\_contig-100\_117.139618SRS013951\_contig\_number\_34532SRS014235\_contig\_number\_6696SRS014459\_contig\_number\_26359SRS014855\_contig\_number\_17694SRS014923\_contig\_number\_33110SRS015065\_contig\_number\_34048SRS015217\_contig\_number\_15764SRS015431\_contig\_number\_12160SRS015578\_contig\_number\_contig-100\_7.183027SRS015663\_contig\_number\_15801SRS016095\_contig\_number\_30269SRS016132\_contig\_number\_570SRS018836\_contig\_number\_24644SRS019068\_contig\_number\_116323SRS019496\_contig\_number\_9068SRS019685\_contig\_number\_27460SRS019693\_contig\_number\_contig-100\_32.42457SRS020328\_contig\_number\_6634SRS042628\_contig\_number\_contig-100\_18.195351SRS043001\_contig\_number\_11037SRS044535\_contig\_number\_22427SRS045645\_contig\_number\_14532SRS047014\_contig\_number\_contig-100\_2205.182405SRS047044\_contig\_number\_15821SRS049712\_contig\_number\_11565SRS049896\_contig\_number\_18163SRS051031\_contig\_number\_26967SRS052697\_contig\_number\_15457SRS054956\_contig\_number\_1865SRS055017\_contig\_number\_32910SRS058723\_contig\_number\_3481SRS063040\_contig\_number\_33929SRS063985\_contig\_number\_27316SRS064757\_contig\_number\_23076SRS076929\_contig\_number\_25086SRS077194\_contig\_number\_31350SRS077335\_contig\_number\_7499SRS077502\_contig\_number\_15191SRS077552\_contig\_number\_27932SRS077641\_contig\_number\_22153SRS098073\_contig\_number\_17855SRS098514\_contig\_number\_97SRS098571\_contig\_number\_31987SRS1041037\_contig\_number\_14232SRS1041129\_contig\_number\_12004SRS1041145\_contig\_number\_33120SRS104197\_contig\_number\_contig-100\_201.3251SRS104400\_contig\_number\_52314SRS104636\_contig\_number\_contig-100\_103.158254SRS1055067\_contig\_number\_4877SRS1055076\_contig\_number\_contig-100\_756.90045SRS142503\_contig\_number\_58297SRS142599\_contig\_number\_16406SRS143085\_contig\_number\_1938SRS143148\_contig\_number\_27059SRS143598\_contig\_number\_6112SRS143991\_contig\_number\_27141SRS144135\_contig\_number\_contig-100\_254.195998SRS144537\_contig\_number\_29383SRS144714\_contig\_number\_contig-100\_24.167976SRS146764\_contig\_number\_33708SRS146812\_contig\_number\_62433SRS147139\_contig\_number\_44618SRS147272\_contig\_number\_1386SRS147766\_contig\_number\_20346SRS147919\_contig\_number\_21465SRS148159\_contig\_number\_47736SRS148196\_contig\_number\_15981SRS148874\_contig\_number\_16675SRS148970\_contig\_number\_contig-100\_11.39533SRS149181\_contig\_number\_30051SRS149879\_contig\_number\_24459SRS893231\_contig\_number\_5485SRS893252\_contig\_number\_838SRS893270\_contig\_number\_19876SRS893285\_contig\_number\_1714SRS893295\_contig\_number\_12031
39485
80
species

species
1
142586

SRS019787\_contig\_number\_9789
